# 异基因造血干细胞移植后耶氏肺孢子菌肺炎20例临床特征

**DOI:** 10.3760/cma.j.cn121090-20240217-00062

**Published:** 2024-09

**Authors:** 瑞 马, 姝婷 常, 晓冬 莫, 萌 吕, 昱 王, 晓辉 张, 兰平 许, 晓军 黄, 于谦 孙

**Affiliations:** 北京大学人民医院，北京大学血液病研究所，国家血液系统疾病临床医学研究中心，造血干细胞移植治疗血液病北京市重点实验室，北京 100044 Peking University People's Hospital & Peking University Institute of Hematology, National Clinical Research Center for Hematologic Disease, Beijing Key Laboratory of Hematopoietic Stem Cell Transplantation, Beijing 100044, China

## Abstract

2014年4月至2022年10月，20例血液病患者在北京大学人民医院接受异基因造血干细胞移植（allo-HSCT）后发生耶氏肺孢子菌肺炎（pneumocystis jirovecii pneumonia, PJP）。男13例（65.0％），女7例（35.0％），中位年龄34（19～60）岁。急性髓系白血病11例（55.0％），急性淋巴细胞白血病4例（20.0％），骨髓增生异常综合征2例（10.0％），慢性粒-单核细胞白血病1例（5.0％），非霍奇金淋巴瘤1例（5.0％），再生障碍性贫血1例（5.0％）。同胞全相合移植3例（15.0％），无关供者移植3例（15.0％），单倍体移植14例（70.0％）。PJP中位发病时间为移植后353（74～1 121）d。主要临床症状为发热、咳嗽、咯痰、呼吸困难。所有患者CT检查均有双肺弥漫磨玻璃影、斑片影、实性结节等感染征象。9例（45.0％）患者需鼻导管吸氧支持，7例（35.0％）患者需呼吸机辅助呼吸。重症感染7例（35.0％），轻、中度感染13例（65.0％）。8例（40.0％）患者发生巨细胞病毒感染，2例患者发生EB病毒感染。所有20例患者均接受复方磺胺甲唑治疗（标准剂量11例，低剂量9例）。19例患者存活，1例患者死亡。

## Abstract (en)

This study included 20 patients with hematological diseases who developed Pneumocystis jirovecii pneumonia（PJP）after receiving allogeneic hematopoietic stem cell transplantation（allo-HSCT）from April 2014 to October 2022 at Peking University People's Hospital. The 20 patients comprised 13 males（65.0％）and seven females（35.0％）, with a median age of 34（19–60）years. Eleven cases（55.0％）of acute myeloid leukemia, four cases（20.0％）of acute lymphocytic leukemia, two cases（10.0％）of myelodysplastic syndrome, one case（5.0％）of chronic myelomonocytic leukemia, one case（5.0％）of non-Hodgkin lymphoma, and one case（5.0％）of aplastic anemia were analyzed. Three cases（15.0％）of HLA-identical sibling hematopoietic stem cell transplantation, three cases（15.0％）of matched unrelated donor hematopoietic stem cell transplantation, and 14 cases（70.0％）of haploid hematopoietic stem cell transplantation were identified. The median onset time of PJP was 353（74–1121）days after transplantation. The clinical symptoms mainly included fever, cough, expectoration, and dyspnea. All patients presented signs of infection based on the CT scan, including bilateral diffuse ground-glass opacities, patchy shadows, and solid nodules. Nine patients（45.0％）required respiratory support via nasal catheter oxygen inhalation, while seven patients（35.0％）required ventilator-assisted breathing. Seven（35.0％）severe infections and 13（65.0％）mild to moderate infections were recorded. Moreover, eight patients（40.0％）were complicated with human cytomegalovirus infection, whereas two patients were complicated with EB virus infection. Furthermore, all 20 patients received treatment with compound sulfamethoxazole（standard dose, 11 cases; low dose, 9 cases）. Furthermore, 19 patients survived and one patient died.

耶氏肺孢子菌肺炎（pneumocystis jirovecii pneumonia, PJP）是由耶氏肺孢子菌感染引起的急性或亚急性肺炎，临床多表现为发热、呼吸困难、干咳、严重低氧血症[1]，多见于人类免疫缺陷病毒（HIV）感染患者等免疫缺陷人群，约1/3发生于非HIV免疫缺陷群体（血液系统恶性肿瘤、实体器官肿瘤、接受免疫抑制剂治疗患者）[2]。近年来非HIV免疫缺陷患者所占比例升高。异基因造血干细胞移植（allo-HSCT）患者由于伴有持久性免疫缺陷，是PJP的主要发生人群。国外数据显示allo-HSCT后PJP的发生率为0.53％～0.63％[3]，未接受复方磺胺甲唑预防治疗患者的病死率为5％～37％，接受复方磺胺甲唑预防治疗患者的病死率为1％～6％[4]。本研究对北京大学人民医院血液科allo-HSCT后PJP患者的临床资料进行回顾性分析，总结allo-HSCT后PJP感染的临床特点，以期为allo-HSCT后PJP的管理提供支持。

## 病例与方法

一、病例资料

本研究为回顾性分析，纳入2014年4月至2022年10月在北京大学人民医院接受allo-HSCT后发生PJP的20例患者。本研究获得北京大学人民医院伦理委员会审查通过。

二、移植方案

移植方案见文献[5]。预处理方案见文献[6]–[7]：阿糖胞苷4 g·m^−2^·d^−1^静脉滴注，−9 d；白消安0.8 mg/kg每6 h 1次静脉滴注，−8～−6 d；环磷酰胺1.8 g·m^−2^·d^−1^静脉滴注，−5 d、−4 d；司莫司汀250 mg/m^2^口服，−3 d；抗胸腺细胞球蛋白（ATG）2.5 mg·kg^−1^·d^−1^静脉滴注，−5 d～−2 d。GVHD的诊断及分级参照MAGIC标准[8]。

三、PJP的诊断

参照欧洲癌症研究和治疗组织/侵袭性真菌感染协作组（EORTC/MSGERC）[9]对非HIV患者PJP的定义进行PJP诊断。确诊PJP患者为显微镜检或免疫荧光技术在组织、支气管肺泡灌洗液（BALF）或痰液中检出耶氏肺孢子菌。临床诊断需同时满足宿主因素、临床特征及微生物证据。其中，宿主因素包括：在诊断前60 d内应用糖皮质激素超过2周，CD4阳性淋巴细胞计数降低，暴露于抗肿瘤、抗炎或免疫抑制治疗，原发性免疫缺陷，恶性血液病，实体器官移植，造血干细胞移植等。临床特征包括：影像学特征（双侧磨玻璃影，实变、小结节或单侧浸润、大叶浸润、结节浸润伴或不伴空洞、粟粒样改变等）和呼吸系统症状（咳嗽、呼吸困难或低氧血症等）。微生物证据包括：至少连续2次血清ß-D-葡聚糖≥80 ng/L；实时定量PCR检测到呼吸道标本中耶氏肺孢子菌DNA阳性。本研究中，宏基因组二代测序技术（mNGS）在BALF中检出耶氏肺孢子菌基因序列也作为临床诊断的病原学依据[10]–[12]。

四、PJP的预防与治疗

移植预处理开始至移植后1个月，予以复方磺胺甲唑［每片含磺胺甲唑（TMP）400 mg、甲氧苄啶（SMZ）80 mg］2片每日2次口服；移植后1～6个月，复方磺胺甲唑4片每周2次口服。

参照欧洲白血病抗感染指南（ECIL）治疗指南[13]进行治疗：首选标准剂量复方磺胺甲唑（TMP 15～20 mg·kg^−1^·d^−1^，SMZ 75～100 mg·kg^−1^·d^−1^），疗程14 d；部分患者因肾功能不全等原因接受低剂量复方磺胺甲唑（TMP<15 mg·kg^−1^·d^−1^，SMZ <75 mg·kg^−1^·d^−1^），疗程14 d。糖皮质激素应用的时机和剂量由临床医师决定[14]。

PJP的疗效评估参考《血液病/恶性肿瘤患者侵袭性真菌病的诊断标准与治疗原则（第六次修订版）》[15]。治疗有效包括完全缓解和部分缓解，治疗无效包括稳定、疾病进展和死亡。

## 结果

一、基线特征

20例移植后PJP患者中，男13例（65.0％），女7例（35.0％）；中位年龄34（19～60）岁。其中，急性髓系白血病11例（55.0％），急性淋巴细胞白血病4例（20.0％），骨髓增生异常综合征2例（10.0％），慢性粒-单核细胞白血病1例（5.0％），非霍奇金淋巴瘤1例（5.0％），再生障碍性贫血1例（5.0％）。移植相关资料详见表1。

**表1:** 20例耶氏肺孢子菌肺炎（PJP）患者的一般特征和移植相关资料

基线特征	结果
性别［例（％）］	
男	13（65.0）
女	7（35.0）
中位年龄［岁，*M*（范围）］	34（19~60）
供者类型［例（％）］	
同胞全相合	3（15.0）
无关	3（15.0）
单倍体	14（70.0）
含ATG预处理方案［例（％）］	
是	17（85.0）
否	3（15.0）
单个核细胞［×10^8^/kg，*M*（范围）］	8.43（6.22~11.44）
CD34^+^细胞［×10^6^/kg，*M*（范围）］	2.1(1.2~6.6)
急性GVHD［例（％）］	3（15.0）
慢性GVHD［例（％）］	4（20.0）
CMV感染［例（％）］	8（40.0）
EBV感染［例（％）］	2（10.0）

**注** ATG：抗胸腺细胞球蛋白；GVHD：移植物抗宿主病；CMV：巨细胞病毒；EBV：EB病毒

二、临床特点

20例患者诊断PJP的中位时间为移植后353（74～1121）d，主要临床症状包括：发热17例（85.0％）、咳嗽13例（65.0％）、咳痰6例（30.0％）、喘憋4例（20.0％）、呼吸困难6例（30.0％）、流涕1例（5.0％）。9例（45.0％）患者需要鼻导管吸氧，7例（35.0％）患者需要呼吸机辅助呼吸。所有患者CT检查均有感染征象：双肺弥漫磨玻璃影13例（65.0％）、斑片影12例（60.0％）、实性结节影7例（35.0％）。病原学证据方面，2例患者通过六胺银染色确诊（BALF、痰液标本各1例）。16例（80.0％）患者G试验阳性，6例（30.0％）患者GM试验阳性。4例患者BALF标本实时定量PCR检测耶氏肺孢子菌DNA阳性（同时G试验阳性），1例患者痰液实时定量PCR检测耶氏肺孢子菌DNA阳性（同时G试验阳性）。10例患者BALF标本中mNGS检出耶氏肺孢子菌序列同时G试验阳性，均满足临床诊断标准。另有3例患者仅BALF标本mNGS检出耶氏肺孢子菌序列。

诊断时20例（100％）患者均处于非粒细胞缺乏状态，7例（35.0％）诊断时合并GVHD，9例（45％）发生病毒感染。

三、治疗与转归

20例患者中，11例（55.0％）在诊断PJP前仍处于复方磺胺甲唑预防治疗中，11例（55.0％）在诊断PJP前有糖皮质激素长期用药史。诊断后有9例（45.0％）患者立即启动标准剂量复方磺胺甲唑治疗，另有11例（55.0％）患者因各种药物不良反应采用低剂量复方磺胺甲唑治疗。15例（75.0％）患者在治疗过程中加用了糖皮质激素。19例（95％）患者病情好转，1例（5％）患者死于感染。

## 讨论

耶氏肺孢子菌是allo-HSCT后的重要感染病原体。EORTC/MSGERC数据显示，PJP的累积发生率在allo-HSCT后1、2、3、5年分别为1.16％、3.32％、3.52％、3.52％。尽管有效的预防可以显著降低感染的发生率，PJP在移植后晚期仍有较高的发生率。高龄、供者类型、慢性GVHD是移植后期PJP感染的独立危险因素[16]。PJP一旦发生，疾病进展较快，如不进行快速准确诊断及治疗，死亡率较高。国内目前缺乏相应流行病学数据。

PJP临床表现缺乏特异性，高度依赖病原学检查。传统病原学检出率较低，实时定量PCR检测呼吸道标本应用越来越广泛，也被EORTC/MSGERC推荐为诊断依据之一。但PCR方法区分耶氏肺孢子菌定植与感染所需的阈值尚存在争议，且在单次检测混合感染中价值有限。作为新型的诊断技术，mNGS在常规抗感染治疗效果不理想时是有潜力的诊断手段[17]。

PJP患者疾病进展快，尽早诊断及及时有效的治疗有助于降低病死率。文献报道33％的患者需要在重症监护病房治疗，病死率为14％[18]。长期使用免疫抑制剂、巨细胞病毒感染、淋巴细胞计数减少、住院期间有创通气是移植后PJP患者死亡相关的危险因素[19]。本组病例中需使用无创呼吸机辅助通气患者占35.0％，与上述文献相似；但病死率（5.0％）明显低于文献报道，其原因可能包括：①本研究样本量较小；②本研究中mNGS诊断病例占比较高，病原菌负荷相对较小；③本研究中病例感染发生时间相对较晚。但上述结果仍需更大样本研究进一步验证。

复方磺胺甲唑是PJP的首选治疗。部分患者由于存在肾功能异常、过敏、胃肠道症状等情况，无法耐受标准剂量治疗。本组患者中9例（45.0％）患者采用低剂量复方磺胺甲唑治疗，治疗效果与标准剂量无显著差异，与文献报道的对于非HIV感染PJP患者采用低剂量TMP/SMZ亦有较好的治疗效果相符[20]。近期一项在血液肿瘤患者中进行的分析也显示，低剂量复方磺胺甲唑治疗在轻中度患者中不影响治疗结果[21]。

糖皮质激素常常用于PJP的治疗，但其应用价值文献报道不一。一项荟萃分析研究显示，糖皮质激素治疗对非HIV感染PJP患者死亡率没有影响，对严重低氧血症患者无获益，且对机械通气次数和持续时间无显著影响[22]。另一大样本研究[23]纳入了1 299例非HIV所致PJP患者（重度低氧血症患者737例，中度低氧血症患者562例），在重度低氧血症患者中，应用糖皮质激素与60天死亡风险相关（*HR*＝0.71，95％*CI* 0.55 ～0.91），可使病死率显著降低（24.7％对36.6％，*P*＝0.006）；而在中度低氧血症患者中，应用糖皮质激素与60天全因死亡率无显著相关性。另有研究显示，糖皮质激素辅助抗PJP治疗的早期应用在患者病死率、住院时间、机械通气需求等方面没有差异[24]。因此，针对不同严重程度的PJP感染，是否应用糖皮质激素及其应用时机、剂量及疗程等方面仍需要进一步研究。

总之，本组病例结果显示PJP常发生于allo-HSCT后晚期，经早期诊断、治疗可获得较好的疗效。上述结论尚需开展多中心临床研究加以验证。
